# Younger ages at risk of Covid-19 mortality in communities of color

**DOI:** 10.12688/gatesopenres.13151.1

**Published:** 2020-06-26

**Authors:** Keith P. Klugman, Solomon Zewdu, Barbara E. Mahon, Scott F. Dowell, Padmini Srikantiah, Kayla F. Laserson, Jordan W. Tappero, Anita K. Zaidi, Trevor Mundel

**Affiliations:** 1 Global Health, Bill & Melinda Gates Foundation, Seattle, WA, 98119, USA; 2 Global Development, Bill & Melinda Gates Foundation, Seattle, WA, 98119, USA

**Keywords:** COVID-19, Africa, Youth, Mortality, Communities of Color

## Abstract

More than 85% of Covid-19 mortality in high income countries is among people 65 years of age or older. Recent disaggregated data from the UK and US show that minority communities have increased mortality among younger age groups and in South Africa initial data suggest that the majority of deaths from Covid-19 are under 65 years of age. These observations suggest significant potential for increased Covid-19 mortality among younger populations in Africa and South Asia and may impact age-based selection of high-risk groups eligible for a future vaccine.

Developed countries, even those overwhelmed for a time by Covid-19 like Italy and the UK, have had most of their deaths among the elderly. In Italy, with one of the oldest populations in the world, only 4% of deaths were in people under age 60 ^1^. This has led to speculation that Africa and South Asia, with younger populations, may be spared most of the mortality of this pandemic. Recent national data from the UK, US and South Africa suggest that this may not be the case.

In the UK, the racial disparity in Covid-19 mortality is clear, with non-white minorities dying at higher rates than whites ^2^. The reasons for this disparity are not yet clear but may include higher rates of exposure due to socio-economic factors reducing ability to social distance, poorer access to or quality of care, and / or higher rates of severe disease among infected persons. In England and Wales, black males are 4.2 times more likely to die of Covid-19 than are white males, and black women are 4.3-fold more likely to die of Covid-19 than white women, in analyses adjusted for differences in age between these communities ^2^.

In addition, England and Wales have reported concerning racial differences in age at death from Covid-19. Although age-specific death rates have not yet been reported for different racial groups, just 9.8% of white patients in England and Wales who died of Covid-19 were under age 65, whereas among those with black ancestry, 24.2% were under age 65, as were 20.9% of those with ancestry from India, Bangladesh or Pakistan ^2^. In sum, the data from the UK ^2^ show both excess mortality, particularly in black communities, and increased proportions of death in younger age groups. This pattern cannot be explained just by younger age structure in these communities. Age is the most important risk factor for Covid-19 mortality in all these communities ^2^. Therefore, in communities with younger age structures it is expected that there would be less total mortality, not more mortality, than in older age structured communities. The excess mortality at younger ages relative to Caucasian people in the UK therefore reflects excess risk—of exposure, of severe disease in infected persons, or both—despite younger age.

Similar concerning data have now been released by the CDC ^3^, showing that, while just 9.7% of white Americans who die from Covid-19 are under 65 years of age, that percentage among black Americans is 24.2%. Among Hispanic Americans 35.6% are less than age 65 at the time of their Covid-19-related death, as are 40.4% of American Indians or Alaska Natives, and 42.9% of Hawaiians or Other Pacific Islanders.

Major comorbidities for Covid-19 mortality, including diabetes, hypertension and chronic lung disease are concentrated in poorer communities, as illustrated in data from across multiple counties in the US ^4^. 

These data thus may pose major problems for both South Asia and Africa, where poverty and lack of access to care may further increase risk of these risk factors for Covid-19 mortality. Major comorbidities that increase the risk for severe Covid-19 disease may explain some of this disparity in mortality. Untreated hypertension and diabetes are increasing rapidly in Africa and South Asia, particularly in urban centers. A 2017 paper surveying a representative sample of 16,287 adults over age 2, in three cities in South Asia--Chennai, Delhi and Karachi--documented that 30.1% of men and 26.89% of women living in those cities had hypertension, with only 1/7 on treatment ^5^. The Covid-19 pandemic portends greater mortality at younger ages in these settings than in rich countries with generally better access to care of comorbid conditions. 

As of the first week of June 2020, the epidemic continues to expand in Latin America, and is gaining significant momentum in both South Asia and Africa. In the data on the first 752 Covid-19 deaths with known age in South Africa, 66.4% were aged less than 65 ^6^. While only 5% of the South African population at their last census were aged 65 or older, fully 26.5% of the population is 40-65 years of age, the age group associated with 58% of deaths ^6^. The majority of deaths in South Africa at this early stage of their epidemic are in the Western Cape, reflecting increased risk of importation from travelers, and the increase in deaths at younger ages is becoming more pronounced each week, as local transmission in poor communities increases ^6^. In the past week the first preliminary data on poor quality of care as a risk for Covid-19 mortality have been presented for the Western Cape in South Africa ^7^, showing increased mortality for people with untreated and poorly treated diabetes, compared to well controlled diabetes. Also, the first data suggesting HIV infection may increase risk for Covid-19 mortality have just been released. These data are not yet published or peer-reviewed, but if they hold up under review, they will suggest significantly increased risks for mortality in younger South Africans consistent with the large fraction of deaths in younger age groups ^6^.

These data raise important questions on implications for access to what are likely to be limited initial quantities of a vaccine. While many factors will determine access to vaccine and specific groups such as first responders may get first access to vaccine, age-based indications for early doses of Covid-19 vaccines will be confounded by these data. Will age-based risk be taken into account to protect the majority of those at risk for age related Covid-19 mortality? This is not only a global question, it also will need to be addressed in the US, as shown in Table 1, where vaccine given at ages 65 years of age and above addresses 90% of the mortality burden in non-Hispanic white people, but only 64% of Hispanic or Latino people and 57% of Hawaiian and other Pacific Islanders.

**Table 1.** US Covid-19 mortality data from the National Center for Health Statistics ^3^. Bold numbers indicate the age group above which 89% or more of covid-19 deaths have occurred.

Age	All	Non-Hispanic White	Non-Hispanic Black	Non-Hispanic Indian or Alaska Native	Non-Hispanic Asian	Non-Hispanic Hawaiian and other Pacific Islander	Hispanic or Latino
All	88243	46965	20288	463	4648	63	14455
≥65	71186 (81%)	**42196 (90%)**	14723 (73%)	276 (60%)	3621 (78%)	36 (57%)	9316 (64%)
≥55	**81772 (93%)**		**18195 (90%)**	363 (78%)	**4282 (92%)**	49 (78%)	11976 (83%)
≥45				**413 (89%)**		**56 (89%)**	**13516 (94%)**

Equitable age-based vaccination strategies will need to consider adjustment for age specific patterns of mortality in some communities.

## Data availability

All data underlying the results are available as part of the article and no additional source data are required.

## References

[1] statista.com/statistics/1105061/coronavirus-deaths-by-region-in-italy/. Data as of 3 June 2020. Reference Source

[2] Office for National Statistics: Coronavirus (COVID-19) related deaths by ethnic group, England and wales: 2 March 2020 to 10 April 2020.2020 Reference Source

[3] National Center for Health Statistics: Weekly updates by select demographic and geographic characteristics. Updated June 3, 2020.2020 Reference Source

[4] Shaw KM Theis KA Self-Brown S: Chronic disease disparities by county economic status and metropolitan classification, behavioral risk factor surveillance system, 2013. *Prev Chronic Dis.* 2016;13:E119. 10.5888/pcd13.160088 27584875 PMC5008860

[5] Prabhakaran D Jeemon P Ghosha S: Prevalence and incidence of hypertension: Results from a representative cohort of over 16,000 adults in three cities of South Asia. *Indian Heart J.* 2017;69(4):434–441. 10.1016/j.ihj.2017.05.021 28822507 PMC5560901

[6] National Institute for Communicable Diseases: Covid-19 sentinel hospital surveillance, South Africa, week 23, 2020.2020 Reference Source

[7] Davies MA on behalf of Western Cape Department of Health: Western Cape: COVID-19 and HIV / Tuberculosis.2020; Accessed 6/17/2020. Reference Source

