# Supplementary material for: Younger ages at risk of Covid-19 mortality in communities of color
Source: Gates Open Res. 2020 Jun 26;4:69. [Version 1] doi: 10.12688/gatesopenres.13151.1 (PMC7360879; doi:10.12688/gatesopenres.13151.1)
Supplement: Peer review history

# Peer review: Reviewer response for version 1

**DOI:** 10.21956/gatesopenres.14345.r28994

**Published:** 2020-07-14

**Authors:** Charles Feldman, Guy Anthony Richards

**Affiliations:** 1 Department of Internal Medicine, Faculty of Health Sciences, University of the Witwatersrand, Johannesburg, Johannesburg, South Africa; 2 Department of Critical Care, Faculty of Health Sciences, University of the Witwatersrand, Johannesburg, South Africa

This article, published on Gates Open Research, discusses the emerging evidence that people of younger age in minority communities are at increased risk of death from COVID-19 when compared with people living in high income countries, where most deaths occur in the elderly.

 This issue is raised by the authors in consideration of the implications this may have on access to what are likely to be limited initial quantities of a vaccine for this infection. The authors further indicate that risk factors for COVID-19 severity and mortality, such as diabetes mellitus, hypertension, and chronic lung disease, are more common in poorer communities; many studies suggest that these are occurring at younger ages. Coupled with poverty, reduced ability to practice social distancing, possible lack of access to care, and resultant poor treatment, this may place younger people in these communities at greater risk of COVID-19 mortality.

 While it is acknowledged by the authors that there will be many factors that will determine access to the vaccine, they suggest that simple age-based recommendations may be confounded by these facts, and if age based recommendations are introduced to protect those at risk of age related COVID-19 mortality, they will need to be adjusted for age-specific patterns of risk factors in certain communities.

 While we would agree with these recommendations regarding age, there also needs to be consideration of the importance of much further research into what the exact factors are that are related to these community-based differences. This includes the additional consideration of the potential role of nutritional deficiencies, as well as obesity, genetic differences such as polymorphisms in the ACE2 receptor that may put some populations at greater risk, as well as our understanding of what treatments are being offered in different communities in cases with active COVID-19 infection. One important understanding that is still urgently needed is what the COVID-19 risk is for people living with HIV (PLWH). Although the authors indicated that the initial data from Cape Town, South Africa, as yet not peer reviewed or published, suggested a greater risk for PLWH, data from a study from Johannesburg, South Africa, of their first 100 COVID-19 cases, suggested that PLWH were not at greater of COVID-19 infection than the general population, and none of the HIV-positive patients in their study had died (Zamparini *et al.*, 2020 ^1^). There have been other studies published that suggest that HIV is not a major risk factor, and knowledge of the real risk associated with HIV infection, as well as the other issues mentioned above, may assist in better stratifying risks in different populations and prioritising them for vaccine use. Perhaps these additional points should be included in the paper.

Are sufficient details of methods and materials provided to allow replication by others?

Yes

Is the rationale for creating the dataset(s) clearly described?

Yes

Are the datasets clearly presented in a useable and accessible format?

Yes

Are the protocols appropriate and is the work technically sound?

Yes

Reviewer Expertise:

Respiratory infections, particularly community-acquired pneumonia, including COVID-19

We confirm that we have read this submission and believe that we have an appropriate level of expertise to confirm that it is of an acceptable scientific standard.

## References

[1] : Clinical characteristics of the first 100 COVID-19 patients admitted to a tertiary hospital in Johannesburg, South Africa. *Wits Journal of Clinical Medicine*.2020;2(2) :105-114 Reference source

# Peer review: Reviewer response for version 1

**DOI:** 10.21956/gatesopenres.14345.r28995

**Published:** 2020-07-10

**Authors:** Arnold S. Monto

**Affiliations:** 1 Department of Epidemiology, University of Michigan School of Public Health, Ann Arbor, MI, USA

There are a number of difficulties in following the age specific impact of Covid-19 in the middle of the pandemic when much of the information is tentative. However, that does not mean that analysis of epidemiologic findings should not be done. In fact, it is essential for planning purposes to use the data that are available, with appropriate caveats. Issues in dealing with data on the differential impact of the pandemic in different racial and ethnic groups in the US include the lack of necessary information in many of the data sources available and also the very spotty nature of viral outbreaks. An example of the latter is the heavy contribution of nursing or care homes in terms of mortality data. Residents are typically older than the population from which they are drawn, and deaths in these outbreaks will skew the age distribution upward. While it is clear in the US that minority groups in major cities were more heavily infected and had worse outcomes, it is not clear why that is the case. As the pandemic evolves, it is becoming more definite that close contact over time is likely to increase likelihood of transmission, which is more common in disadvantaged communities. They also have more underlying conditions at a younger age, so the combination will lead to higher mortality at a younger age in these populations. The situation in the UK is similar in many respects. Of concern there is the fact that it is not clear how care home data are being handled. Also, there is significant suggestion that socioeconomic status is driving some of the racial/ethnic differences. There is even one report of little difference in that factor in a more affluent suburb of London, a report which is at odds with other reports of racial differences in health care workers and other populations.

 The current data note examines distribution of deaths by age initially in the UK and in the US. It takes the position that, since deaths are occurring at lower ages in minority communities in these countries, it will probably also be the case in Africa and parts of Asia. The evolving situation in the Western Cape of South Africa is given in support.  I agree with this conclusion but am concerned that there will also be another factor at play in following the severity of the outbreak in places which do not have the level of intensive care present in the US and UK, necessary to ensure survival. There also seems to be a systematic underreporting of Covid mortality going on in some of these countries, driven by deaths at home not being properly attributed and others in hospital being counted as being caused by the comorbidities and not by the SARS-CoV-2 virus. Those issues are not easily solved but should be factored into estimates of impact.

 The note mainly uses proportions rather than estimated incidence or age specific mortality rates. That is based on the data available, but it might be more reflective of impact on, for example, the health care system to estimate what that would translate to in actual mortality rates.

Are sufficient details of methods and materials provided to allow replication by others?

Yes

Is the rationale for creating the dataset(s) clearly described?

Yes

Are the datasets clearly presented in a useable and accessible format?

Yes

Are the protocols appropriate and is the work technically sound?

Yes

Reviewer Expertise:

NA

I confirm that I have read this submission and believe that I have an appropriate level of expertise to confirm that it is of an acceptable scientific standard.
